# Prevalence of Canine Obesity, Obesity-Related Metabolic Dysfunction, and Relationship with Owner Obesity in an Obesogenic Region of Spain

**DOI:** 10.3389/fvets.2017.00059

**Published:** 2017-04-25

**Authors:** J. Alberto Montoya-Alonso, Inmaculada Bautista-Castaño, Cristina Peña, Lourdes Suárez, M. Candelaria Juste, Asta Tvarijonaviciute

**Affiliations:** ^1^Internal Medicine, Faculty of Veterinary Medicine, Research Institute of Biomedical and Health Sciences (IUIBS), Las Palmas de Gran Canaria University, Las Palmas, Spain; ^2^Department of Clinical Sciences, Faculty of Medicine, Research Institute of Biomedical and Health Sciences (IUIBS), Las Palmas de Gran Canaria University, Las Palmas, Spain; ^3^Department of Animal Medicine and Surgery, Faculty of Veterinary Medicine, Universidad Autónoma de Barcelona, Barcelona, Spain

**Keywords:** body condition, body mass index, dog, epidemiology, metabolic syndrome, obesity, owner–dog relationship

## Abstract

The main objective of this study was to evaluate the prevalence of canine obesity and obesity-related metabolic dysfunction (ORMD) in the obesogenic area in Spain. The prevalence of overweight/obesity among owners of obese pets was also evaluated. In the sample population studied (93 client-owned dogs), 40.9% of dogs presented obesity (body condition score 7–9/9), 40.9% of dogs presented hypertension, 20.4% of dogs presented fasting hypertriglyceridemia, 20.4% fasting hypercholesterolemia, and 5.4% of dogs presented fasting hyperglycemia. The overall prevalence of ORMD was of 22.6%. Seventy-eight percent of overweight/obese owners had overweight/obese dogs (*P* < 0.001) including all dogs diagnosed with ORMD. In conclusion, in the studied obesogenic region of Spain, the prevalence of canine obesity and ORMD was shown to be elevated and related to the presence of overweight/obesity in owners. All dogs with ORMD were owned by overweight/obese persons. These results provide new inputs for future studies highlighting the relationship between owner and pet obesity and indicating the need of further efforts to control and reduce obesity prevalence in both.

## Introduction

Obesity is defined as an accumulation of excessive amounts of adipose tissue in the body, and it is the most common nutritional disorder in companion animals. Obesity is usually the result of either excessive dietary intake or inadequate energy utilization, which causes a state of positive energy balance (1). In humans, the medical importance of obesity lies in the effect on mortality and morbidity of associated diseases. Similarly, obesity has detrimental effects on the health and longevity of dogs (1, 2). Furthermore, different studies indicate that, similarly as occurs in human medicine, the prevalence of overweight/obesity in the canine population is increasing (1, 3).

The Canary Islands is considered to be an obesogenic area, since people there exhibits one of the highest excess weight rates in Spain (4) with prevalence of more than 53% of overweight/obese adults (5). Furthermore, the prevalence of metabolic syndrome (MetS) among adults in the Canary Islands is high reaching up to 24.4% (6). However, no data about prevalence of canine obesity and obesity-related metabolic dysfunction (ORMD) are available. For this reason, the main objective of this study was to evaluate the prevalence of canine obesity and ORMD in the Gran Canaria Island. The secondary objective was to evaluate the possible relationship between owner and pet-dog obesity.

## Materials and Methods

This study was a descriptive observational multicentre study. Dogs were recruited for and assessed during routine veterinary visits in 10 veterinary clinics/hospitals representative of the whole island. All dogs were healthy, with exception of the presence of obesity, as determined by routine examination.

Body condition score (BCS) was assessed using a 9-point scale (7). The BCS was determined by one of the authors L.S. in all cases in order to avoid possible bias. Blood samples were collected after at least 12-h fasting from a jugular or saphenous venipuncture into tubes containing heparin (LH/Li Heparin, Aquisel). The time between the blood sample collection and analyses was <30 min in all cases.

Previously described criteria for canine ORMD were used in this study. In brief, for a dog to be classified as presenting ORMD, the dog had to present obesity (BCS 7–9/9) and any two of the followings: triglycerides (Tg) >200 mg/dL (2.3 mmol/L), total cholesterol (TChol) >300 mg/dL (7.8 mmol/L), systolic blood pressure >160 mmHg, and/or fasting plasma glucose >100 mg/dL (5.6 mmol/L), or previously diagnosed type 2 diabetes mellitus (8).

Blood pressure was assessed using the oscillometric method (memoPRINT^®^) with an appropriately sized cuff applied on the front part of the left foreleg, medioproximal between the elbow and carpal joint (sensor over radial artery), with the patient lying down upright in ventral recumbent position (dog in a relaxed position). Three measures were taken every 10 min.

Total cholesterol, Tg, and glucose were measured using enzymatic commercially available methods in the auto-analyzer (SPOTCHEM EZ SP-4430^®^, Arkray, Inc., Kyoto, Japan).

Body mass index (BMI) was calculated as weight/height^2^ (in kilogram per meter square) for all owners. The clinical evaluation of the owners was carried out by I.B.C. in all cases. Weight and height were measured during the first visit to the veterinary medical centers, with subjects lightly clothed and without shoes on a Roman balance, SECA model 712 with a capacity of 200 kg in 100 g. Height was measured without shoes using a metered scale SECA 221 with a range of 6–230 cm in divisions of 1 mm. Owners were considered overweight/obese if their BMIs were >25 kg/m^2^ (9).

Data analyses were performed using SPSS 19.0 (SPSS Inc., Chicago, IL, USA). Descriptive statistical analysis included the calculation of means, SDs, and proportions. The Chi-square test was performed to compare proportions. In all the cases, the significance level was established at *P* < 0.05.

## Results

Finally, 93 owners agreed to participate in the study. The owners were 51 men and 42 women between 16 and 70 years old with the BMI between 20 and 44 (Table 1). Although no statistically significant differences were observed between men and women in terms of age or BMI, Chi-square analysis revealed significant differences between the two genders when these were subdivided according to the BMI into lean (BMI ≤20), normal (BMI 20–25), and overweight/obese (BMI ≥25) (*P* < 0.05).

**Table 1:** **General characteristics of the participants**.

	Men (*n* = 51)	Women (*n* = 42)	*P*
Age	38.4 ± 16.4	38.4 ± 14.1	0.798^a^
Body mass index (BMI)	27.3 ± 6.8	26.0 ± 5.7	0.628^a^
≥20	12% (*n* = 6)	5% (*n* = 2)	0.022^b^
20–25	39% (*n* = 20)	57% (*n* = 24)	
≥25	49% (*n* = 25)	38% (*n* = 16)	

*^a^Mann–Whitney test*.

*^b^Chi-square test*.

Dogs were between 2 and 14.7 years (mean ± SD, 6.77 ± 3.36 years) old. Thirty-two dogs were males (34.4%), of which 96% were entire, and 61 dogs were females (65.6%), of which 85% were entire. Twenty-three dogs were of mixed breeds and 70 dogs were from 25 recognized breeds, most commonly Yorkshire Terrier, German Shepherd, Miniature Poodle, Cocker Spaniel, Bulldog, and Canarian Dogo. All dogs were fed commercial pet food and received canine snacks and leftovers of kitchen/table scraps.

In the sample population studied, the most frequently present ORMD criteria were obesity and hypertension (41% in both cases), followed by fasting hypercholesterolemia and hypertriglyceridemia (20.4% in both cases), and hyperglycemia (5.4%) (Table 2).

**Table 2:** **The frequency of the ORMD criteria in studied population**.

ORMD criteria	Number of animals
Obesity: BCS 7–9/9	38/93
Hypertension: SBP >160 mmHg	38/93
Hypercholesterolemia: TChol >300 mg/dL (7.8 mmol/L)	19/93
Hypertrigliceridemia: Tg >200 mg/dL (2.3 mmol/L)	19/93
Hyperglycemia: glucose >100 mg/dL (5.6 mmol/L)	5/93

*ORMD, obesity-related metabolic dysfunction; SBP, systolic blood pressure; TChol, total cholesterol; Tg, triglycerides; BCS, body condition score*.

Consequently, in the sample of dogs studied, the prevalence of 22.6% (21/93) of a MetS was observed. No statistically significant differences between dogs presenting and not presenting MetS were found in terms of sex (*P* = 0.380) or age group (*P* = 0.460), classified as being younger or older than 6 years of age.

Relationship between presences or not of overweight/obesity among owners and their pet-dogs obesity is presented in Table 3. Seventy-eight percent of overweight/obese owners had overweight/obese dogs (*P* < 0.001); including all dogs diagnosed with ORMD. Furthermore, owners with BMI ≥25 had younger dogs than those with BMI <25.

**Table 3:** **Owner and pet-dogs data related to presence [body mass index (BMI) ≥ 25] or absence (BMI < 25) of overweight/obesity**.

Group	BMI < 25 (*n* = 52)	BMI ≥ 25 (*n* = 41)	*P*
BMI	22.2 ± 1.3	32.5 ± 5.4	<0.001^a^
Gender (M/F)	26/26	16/25	0.291^b^
Age (years)	37.9 ± 14.6	39.0 ± 16.3	0.873^a^
**Pet-dog data**			
Normal/obese	46/6	9/32	<0.001^b^
Obesity-related metabolic dysfunction	0	21	
Age group: <6 years/>6 years	16/36	25/16	0.004^b^

*^a^Mann–Whitney test*.

*^b^Chi-square test*.

## Discussion

Canine obesity is considered as a major health issue in most developed countries nowadays for its contribution to the development of diseases, as well as decreased lifespan (2). A number of studies have been published reporting the prevalence of the canine obesity in different countries, including France, England, the Netherlands, USA, Australia, China, and among others (10, 11). However, no studies have been reported evaluating the prevalence of canine obesity in Spain.

In this study, the prevalence of the canine obesity in the Canary Islands, Spain, was found to exceed 40% of the evaluated population. These findings are in accordance with those reported in recent obesity prevalence studies that found it to be 41.1% in Australia and 44.4% in China (3, 10). On the other hand, a considerably lower incidence (18.6%) of canine obesity was reported by Corbee (12) among show dogs, probably due to the fact that these dogs needed to be in a good condition for the award achievement.

More than 22% of dogs included in this study were classified as having the ORMD. However, if the presence of ORMD is calculated only among obese dogs, the percentage overpasses 55.3. The observed ORMD prevalence is twice as high as previously reported in an obese dog population in England (8). The multiple factors contributing to obesity could be responsible for the observed differences in prevalence. However, the most important causes of canine obesity, and thus ORMD, were considered as owner-related (12). Thus, the observed prevalence of canine ORMD in this obesogenic area, where quite similar MetS prevalence (of 24.4%) was reported in humans in the same study region, the Canary Islands results (6), could be related with the local nutritional customs. This hypothesis would be also supported by our observations that overweight/obese owners had significantly more overweight/obese dogs and that all dogs with ORMD were owned by overweight/obese persons. One of the limitations of this study was that the presence of MetS could not been evaluated in persons and thus no possible relationships could be determined among occurrence of MetS in dogs and their owners.

In humans, the presence of MetS is considered as a high risk for suffering from cardiovascular diseases and diabetes mellitus (9, 13). However, currently pathogenetic mechanisms and the health significance for dogs, in terms of disease associations and outcomes of weight loss, are not known and need to be evaluated, although it was described that canine ORMD courses with decreased circulating adiponectin, the protein that possess insulin-sensitizing and anti-inflammatory properties (8). Thus, the hypoadiponectinemia could be associated with the increased risk to suffer obesity-related diseases in dogs (8) as it was described in human medicine (14). Furthermore, obese dogs with ORMD showed altered levels of proteins involved in lipid metabolism, immune response, and antioxidant status (15).

This work has a number of limitations including the lack of the information related with the eating/feeding and exercising behavior of the participants, although these were not the objectives of the present study. Furthermore, because of the relatively low number of dogs recruited together with the large variability in ages and breeds, the results have to be interpreted with caution. Nevertheless, these present a true clinical picture of a small Island. However, further long-scale studies would be needed to further confirm the relationship between owner and pet-dog obesity and to identify causal factors for both species.

In conclusion, in the studied obesogenic region of Spain, the prevalence of canine obesity and ORMD were shown to be elevated reaching 40 and 22%, respectively. Overweight/obese owners had significantly more overweight/obese dogs than the lean/normal weight persons; and all dogs with ORMD were owned by overweight/obese persons. These results provide new inputs for future studies highlighting the relationship between owner and pet obesity and indicating the need of further efforts to control and reduce obesity prevalence in both.

## Ethics Statement

The study principles were accepted by the Research Unit of the Veterinary Medicine Service of Las Palmas de Gran Canaria University and were in accordance with good clinical practice and with the guidelines of the Helsinki Declaration for studies conducted using human subjects and in accordance with deontological rules and the existing legislation on animal protection in Spain (law RD53/2013). Informed consents were also signed by the owners. Patients’ data were codified to guaranty anonymity.

## Author Contributions

JM-A and IB-C designed the study, performed data analysis and interpretation, and drafted, revised, and approved the manuscript. CP and LS performed sample collection and analysis. AT, JM-A, and IB-C drafted the work and revised critically. All authors approved the final version.

## Conflict of Interest Statement

The authors declare that the research was conducted in the absence of any commercial or financial relationships that could be construed as a potential conflict of interest.
